# Protocol for stage 2 of the GaP study (genetic testing acceptability for Paget's disease of bone): A questionnaire study to investigate whether relatives of people with Paget's disease would accept genetic testing and preventive treatment if they were available

**DOI:** 10.1186/1472-6963-8-116

**Published:** 2008-05-29

**Authors:** Anne L Langston, Marie Johnston, Jill Francis, Clare Robertson, Marion K Campbell, Vikki A Entwistle, Theresa Marteau, Graeme MacLennan, John Weinman, Marilyn McCallum, Zosia Miedzybrodska, Keith Charnock, Stuart H Ralston

**Affiliations:** 1 Edinburgh Clinical Trials Unit, University of Edinburgh, Queens Medical Research Institute, Room E1.16, 47 Little France Crescent, Edinburgh, EH16 4TJ, UK; 2 Health Services Research Unit, University of Aberdeen, Floor 2, Health Sciences Building, Foresterhill, Aberdeen, AB25 2ZD, UK; 3 Social Dimensions of Health Institute, Universities of Dundee and St Andrews, 11 Airlie Place Dundee, DD1 4HJ, UK; 4 Psychology Department (at Guy's), Health Psychology Section, Psychology and Genetics Research Group, 5th Floor Thomas Guy House, Guy's Campus, London Bridge, London, SE1 9RT, UK; 5 Health Psychology Section, Institute of Psychiatry, 5th Floor Thomas Guy House, London Bridge, SE1 9RT, London, UK; 6 National Association for the Relief of Paget's Disease, 323 Manchester Road, Walkden, Worsley, Manchester, M28 3HH, UK; 7 Clinical Genetics, Argyll House, NHS Grampian, Forsterhill, Aberdeen, AB25 2ZR, UK; 8 Lay adviser, contact via National Association for the Relief of Paget's Disease, 323 Manchester Road, Walkden, Worsley, Manchester, M28 3HH, UK; 9 Molecular Medicine Centre, University of Edinburgh, Western General Hospital, Edinburgh, EH4 2XU, UK

## Abstract

**Background:**

Paget's disease of bone (PDB) disrupts normal bone architecture and causes pain, deformity, deafness, osteoarthritis, and fractures. Genetic factors play a role in PDB and genetic tests are now conducted for research purposes. It is thus timely to investigate the potential for a clinical programme of genetic testing and preventative treatment for people who have a family history of PDB. This study examines the beliefs of relatives of people with PDB. It focuses particularly on illness and treatment representations as predictors of the acceptability and uptake of potential clinical programmes. Illness representations are examined using Leventhal's Common Sense Self-Regulation Model while cognitions about treatment behaviours (acceptance of testing and treatment uptake) are conceptualised within the Theory of Planned Behaviour.

**Methods/Design:**

A postal questionnaire of non-affected relatives of people with Paget's disease. The sample will include relatives of Paget's patients *with *a family history of Paget's disease and relatives of Paget's patients *without *a family history of Paget's disease. The questionnaire will explore whether a range of factors relate to acceptability of a programme of genetic testing and preventive treatment in relatives of Paget's disease sufferers. The questionnaire will include several measures: illness representations (as measured by the Brief Illness Perceptions Questionnaire); treatment representations (as measured by Theory of Planned Behaviour-based question items, informed by a prior interview elicitation study); descriptive and demographic details; and questions exploring family environment and beliefs of other important people.

Data will also be collected from family members who have been diagnosed with Paget's disease to describe the disease presentation and its distribution within a family.

**Discussion:**

The answers to these measures will inform the feasibility of a programme of genetic testing and preventive treatment for individuals who are at a high risk of developing Paget's disease because they carry an appropriate genetic mutation. They will also contribute to theoretical and empirical approaches to predicting diagnostic and treatment behaviours from the combined theoretical models.

## Background

### The need for this research

Over recent years, advances in human molecular genetics have resulted in the identification of polymorphisms and mutations in several genes that cause or predispose to diseases such as cancer, neurodegenerative conditions and inborn errors of metabolism. For some of these conditions, such as Huntington's disease and Muscular Dystrophy, it is possible to offer a genetic test that will give information on the probability of the disease occurring. For other diseases, such as hypercholesterolaemia and haemochromatosis, identification of genetic susceptibility can be used as a risk factor to inform management strategies and treatment decisions in a similar way to other clinical risk factors.

The process of exploring the feasibility of new biomedical developments includes the likely uptake of new tests and treatments.

For example, offering a test to inform on the probability of the disease occurring may be somewhat unattractive to patients without the prospect of an effective treatment. In order to ascertain whether biomedical research will be translated into health care practice, understanding and predicting behaviour is of paramount importance. Translation of genetic knowledge about Paget's disease into practice requires information on the following:

(i) Are people with Paget's disease willing to let their relatives to know of their diagnosis?

(ii) Are people who are genetically related to people with Paget's disease identifiable and happy to be contacted?

(iii) Are medical practitioners likely to refer relatives for genetic testing?

(iv) Are these relatives likely to take a test?

(v) if such a test is positive, are medical practitioners likely to prescribe preventive treatment? and

(vi) Are the relatives likely to adhere to such treatment?

This study concerns points (i), (ii), (iv) and (vi) in this sequence.

Thus, this study aims to test the feasibility of translating genetic knowledge into practice by examining the acceptability of offering genetic testing, followed by therapeutic intervention, to relatives of people with a genetic disease. The study will use established models of social cognition within the field of behavioural science to achieve this. This study will focus on Paget's disease of bone, for which genetic mutations have been identified, and potential effective treatments exist.

### Paget's disease of bone

Paget's disease of bone (PDB) is a common disorder that affects about 2% of individuals over the age of 55 in the UK [1]. It is characterised by focal increases in osteoclastic bone resorption, coupled to increased and disorganised bone formation affecting one or more bones throughout the skeleton. Some patients are asymptomatic, but others develop complications such as bone pain, bone deformity, pathological fracture, deafness and secondary osteoarthritis [1]. These complications cause loss of mobility and independence, and adversely affect quality of life [2]. Genetic factors play an important role in PDB and in 15–40% of families, the disease is inherited as an autosomal dominant trait [3-5]. Mutations have now been identified in four genes that predispose to PDB and related diseases (reviewed by Daroszewska & Ralston [6]) but the most important of these is *SQSTM1 *which is a common cause of classical Paget's disease. About 40% of patients with a familial PDB in the UK carry *SQSTM1 *mutations as do 9% of patients with "sporadic" PDB [7,8]. The proportion of SQSTM1 mutation carriers is higher in the French Canadian population where 50% of familial PDB patients and 20% of "sporadic" patients have *SQSTM1 *mutations [9].

Individuals with *SQSTM1 *mutations are severely affected. The age at diagnosis is about 10 years younger and the disease is more extensive than in PDB patients without *SQSTM1 *mutations [7]. Twelve different mutations of *SQSTM1 *have been described in patients with PDB and all of these cluster in or around the ubiquitin associated (UBA) domain [10].

Functional studies have shown that *SQSTM1 *mutations cause loss of ubiquitin binding [10], suggesting that this may be a unifying mechanism by which the disease occurs, although research is still ongoing to determine how this leads to osteoclast activation.

A few examples have been recorded where carriers of *SQSTM1 *mutations have not developed PDB, even by the seventh decade [9]. This has led to the suggestion that environmental factors such as measles virus infection might interact with *SQSTM1 *mutations to cause PDB [11]. The contribution of viral infection to the pathogenesis of PDB remains controversial, however [12,13]. In any case, irrespective of the possible contribution of environmental factors, between 80%–90% of *SQSTM1 *carriers develop PDB by the sixth or seventh decade [7,9,14]. Importantly, mutations of *SQSTM1 *are highly specific for PDB and have not been found in unaffected age matched controls derived from the general population in any of the studies that have been performed so far [9,14-17]. Progress in understanding the role of *SQSTM1 *in the pathogenesis of PDB has been accompanied by major advances in treatment of the condition.

In view of this, it is timely to investigate the clinical potential for a programme of genetic testing and preventive treatment for patients who carry *SQSTM1 *mutations, in an effort to prevent or delay the development of Paget's disease.

### Prospects for preventing Paget's disease

Aminobisphosphonates such as Risedronate, Pamidronate and Zoledronate have emerged as highly effective agents for reducing bone turnover and treating bone pain in patients with Paget's disease [18]. Recent studies have shown that a single infusion of the fourth generation aminobisphosphonate, Zoledronic acid, can restore alkaline phosphatase levels to normal in about 90% of cases [19]. In the vast majority of these patients, alkaline phosphatase levels have remained normal for 24 months after the infusion [20]. There is also evidence to suggest that standard courses of treatment with intravenous Pamidronate and oral Risedronate can suppress bone resorption in active Paget's disease for up to two years [21,22].

This raises the possibility that patients who carry *SQSTM1 *mutations, and who are at high risk of developing Paget's disease, could be offered prophylactic therapy, in an attempt to prevent the disease occurring, or to prevent complications developing. With the existence of effective treatment that could prevent the onset of disease, we need to explore the acceptability of genetic testing followed by preventive treatment.

### Acceptability of genetic testing and preventive treatment

In light of these developments in Paget's disease, it is timely to investigate whether genetic testing and preventive treatment are acceptable to the relatives of Paget's disease patients. 'Acceptability' has been conceptualised to mean the following behaviours:

• whether relatives of people with Paget's disease would accept such a test if it were offered;

• whether they would accept preventive treatment for Paget's disease, if it were recommended by doctors; and

• if so, what type of treatment (e.g. tablet or injection) they would be prepared to accept, if it were offered.

We have taken a theoretical approach to exploring acceptability by utilising the construct of intention toward the key behaviours contained within recognised social cognition models [23]. Intention to engage in each of the above three behaviours are our measures of acceptability. Within social cognition models, intention is a strong predictor of behaviour. While it is recognised that there is a gap between intention and actual behaviour [24] i.e. intenders do not always carry out the behaviour, it is very rare for non-intenders to do so [25]. In addition, the gap between other cognitions and behaviours is even greater [26]. For example, attitudes to the tests and proposed treatments i.e. the extent to which an individual is favourable toward them, are likely to be less predictive of subsequent behaviours than intentions to engage in the behaviour of accepting tests and treatment, as shown by Fishbein's seminal work in the 1960s.

### Possible factors that could affect acceptability of genetic testing and preventive treatment

Nevertheless, evidence from other areas of genetic testing suggests that patients who say they wish to undertake genetic testing may not actually take up the offer of such a test.

In Huntington's disease, for example, prior to the development of a genetic test, individuals at risk declared a much higher intention of taking a test than the actual numbers taking the test when it became available [27]. Although some people who stated having the intention to take a test did not actually act on this intention, none of those people who did not hold this intention actually took the test. Measuring intentions is, therefore, important as it can very accurately identify those who will definitely not take a test.

Cognitions about genetic conditions in general can also influence acceptability of genetic tests. For example, studies have shown that there is a widespread belief that genetic conditions are not treatable. Such beliefs might affect the acceptability of screening for and treatment of genetic conditions. However, the additional offer of effective preventive treatment can make genetic testing more acceptable [28]. It is, therefore, important to understand the complex issues surrounding acceptability and uptake prior to the development of new technologies to maximise the efficiency of health service provision.

#### Beliefs

Evidence from other, non-genetic, conditions, for which there are effective treatments, demonstrates that patients' cognitions may affect their intentions and therefore uptake of treatment. Two groups of cognitions, representations about illness; and beliefs about behaviours that constitute uptake of screening and treatment are likely to be influential.

#### Illness representations

Illness representations describe how people see their illness. This is most clearly outlined in the Common Sense Self-Regulation Model (CSSRM) [29]. The term 'self-regulation' is defined here as 'processes of goal setting and goal attainment' in response to a threat to health. The five key beliefs that are the components of illness representations are:

• *identity *(e.g. the 'label', identifying symptoms);

• *cause *(e.g. stress, genetics);

• *consequences *(e.g. activity limitations, loss of wages);

• *timeline *(e.g. acute, fluctuating, progressing);

• *cure/control *(e.g. medication, rehabilitation exercises, prior perceived risk of developing the disease);

The model proposes that it is these perceptions, rather than the medical representations, that guide the person's self-regulating efforts. In addition, the CSSRM identifies the *emotional representation *(e.g. frustrating, frightening) as a separate dimension of representation. Based on this model, Weinman and colleagues [30] have developed a measure, the Illness Perceptions Questionnaire (IPQ), to assess these representations and have found that they are predictive of uptake and response to a wide variety of treatments, including the bone disease Rheumatoid Arthritis [31]. Clearly, such perceptions may influence uptake of a new treatment offered to families with Paget's disease. Indeed, Marteau has shown that beliefs affect the decision to accept a genetic test [28].

Moss-Morris et al [32] have subsequently developed a revised version of the Illness Perception Questionnaire (IPQ-R) to include illness *coherence *(e.g. the condition is understandable versus confusing). A brief version of the IPQ-R has also been developed by Broadbent et al [33]. The Brief IPQ provides a rapid assessment of illness perceptions that is quick and easy for participants to complete.

Paget's disease has a highly variable presentation: age at onset, severity of symptoms and impact on sufferers' lives; and individuals might vary in their response to an offer of testing and treatment depending on the disease history within their family. Using the World Health Organisation International Classification of Functioning, Disability and Health model of health components [34], one might expect that perceptions about all three components (impairment, activity limitations, and participation restrictions) might affect beliefs about the identity, consequences and timeline of the condition.

#### Treatment representations

Treatment representations describe how people perceive potential treatments for their disease. There is considerable evidence that patients' beliefs about taking treatments (e.g. ease of access, burden, perceived likelihood of successful consequences) influence uptake and adherence [35]. A programme of genetic testing and treatment requires considerable behavioural input from the individual and their representations of what is involved rather than the objective programme of treatment, are likely to determine their willingness to participate.

### Cognitions about the behaviours involved in uptake in uptake

**Figure 1:**
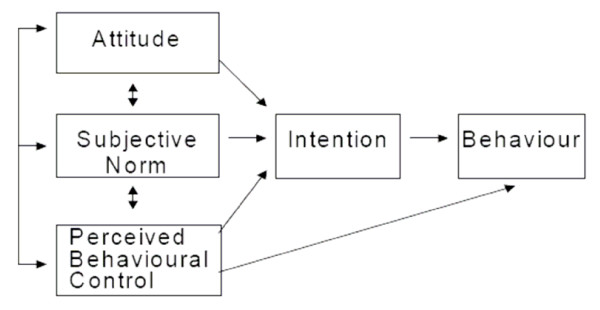
Diagrammatic representation of the Theory of Planned Behaviour.

The Theory of Planned Behaviour (TPB) is a theoretical model (see Figure 1), which predicts intentions and behaviour from people's beliefs about the consequences, social pressures and perceived control over the behaviour, including uptake of testing and uptake of treatment [36]. The TPB proposes that intention to perform a given behaviour is shaped by a person's attitude, subjective norms and perceived behavioural control relating to that behaviour and gives clear guidance on how to operationalise these constructs.

### Attitude

A person's attitude to a behaviour (i.e. whether they evaluative it positively or negatively) is shaped by their behavioural beliefs (beliefs about the consequences of the behaviour e.g. whether it will improve or prevent them having Paget's disease), and their outcome evaluations (the positive or negative features of the consequences of the behaviour e.g. whether they consider avoiding Paget's disease to be important).

### Subjective Norms

A person's own perception of the beliefs and practices of significant others (individuals or groups) as to whether these significant others would approve or disapprove of the behaviour, and the person's own motivation to comply with these pressures. Subjective norms are shaped by beliefs concerning how socially desirable or undesirable it is to perform the behaviour e.g. whether they believe their relatives would wish them to have genetic testing.

### Perceived Behavioural Control

Beliefs about factors that make it easy or difficult for a person to enact the behaviour combined with beliefs about the power of those factors to enable them to enact the behaviour. Control beliefs can be internal (e.g. information, skills, abilities) or situational (e.g. opportunities, barriers, dependence on others) e.g. whether the individual believes that genetic testing is possible for them.

### Intention

Intention is the index of acceptability of genetic testing and treatment for this study. It is the strength of motivation to enact the behaviour. The model describes how people are more likely to engage in attractive behaviours over which they have high levels of control. Behaviours that carry high levels of intention and perceived behavioural control should produce strong motivation to actually enact the behaviour. It is, therefore, possible to produce a questionnaire based on the TPB model to predict intention to perform a given behaviour, such as uptake of a genetic test or preventive treatment, i.e. acceptability.

#### The nature of the treatment and treatment offer

The nature of the treatment, and the way in which the treatment is offered, may affect acceptability. One needs to consider not only how to invite the affected individual to 'involve' their family, but also how to introduce the programme to invited family members. Hardeman *et al *[37] in the *Pro-Active *Programme, have developed an interview for families of people with Type 2 diabetes that incorporates the offer of the intervention for offspring.

This interview was based on the data from interviews used to develop questionnaires for the Theory of Planned Behaviour (TPB) addressing beliefs about involving the offspring in the programme. It has proved very effective in rendering the programme acceptable.

In addition, the treatment offered for Paget's disease could be in tablet or infusion form, and could be administered in a range of environments. All of these factors may influence the acceptability of a programme of genetic testing and preventive treatment and will be explored in this study.

#### Demographic and health characteristics

**Figure 2:**
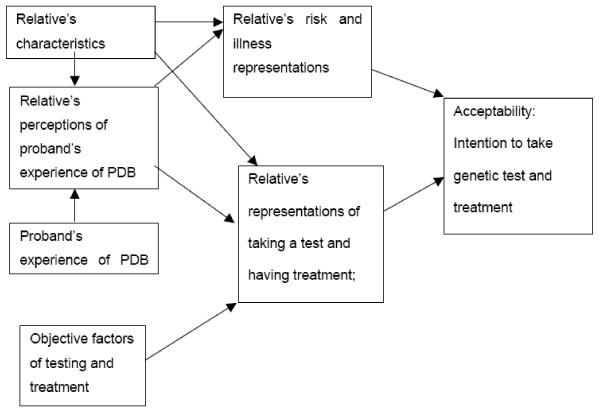
A model of the relationship between explanatory and dependent variables.

Demographic and health-status characteristics may also influence people's illness perceptions as well as their beliefs about the behaviours involved and hence the acceptability of treatment. For example, someone who is older may think they are less at risk if they have passed the age of onset of Paget's disease in the affected person; or they may think they are nearing the time when the disease might affect them; or they may think they are less likely to benefit. Closeness to the affected person may make them view the condition as more or less serious, and they may be influenced by how the affected person copes with the illness. Furthermore, one might expect there to be more agreement within members of one family than between families due to their shared exposure to the condition as well as their opportunity to discuss and influence each other (Figure 2).

We will therefore investigate how Paget's disease has affected the proband, using the three health components of the International Classification of Functioning [34] to characterise the consequences of the disease, i.e. we will assess impairment of body functioning and structure, activity limitations and participation restrictions.

### Scientific value of this study

This project takes advantage of recent advances in knowledge about the molecular-genetic basis of PDB, with advances in therapeutics to address an important clinical question that is of relevance to patients with Paget's disease and their families. The project provides added value in that modern techniques and models in behavioural science will be applied to a specific issue in the treatment of Paget's disease, while at the same time investigating more general theories of human behaviour. Using the Common Sense Model of Self-Regulation, we will investigate how genetic information and preventive opportunities affect self-regulation via illness representations and, using the Theory of Planned Behaviour, how the behaviour of uptake of the offer of genetic testing and treatment is predicted by the constructs found to predict behaviour in other settings. Thus, the findings will be relevant to these theories. They will also contribute to ongoing work to integrate these two models in understanding cognitions and behaviour related to illness threats [38].

In addition, the knowledge gained from this investigation would be relevant to other musculoskeletal diseases such as osteoporosis, which also have a strong genetic component, and which can be prevented by bisphosphonates and hormone replacement therapy. With completion of the human genome project, and advances in human molecular genetics, it is probable that DNA testing with single genetic markers or combinations of markers will be offered for a wide range of other chronic diseases for which effective treatments are available. The results of the present research project will also be of direct relevance to the development of programmes of genetic testing and preventive intervention for these conditions.

### Study Aim

The aims of this study are to understand factors that might influence the acceptability of an offer of a programme of genetic screening and preventive treatment to families of Paget's disease sufferers.

The specific research questions are:

**Stage 1**. (already completed [39])

a): What do individuals with Paget's disease think would influence the involvement of their relatives in a programme of genetic testing and preventive treatment? and;

b) What do relatives of Paget's disease sufferers think would influence them in accepting an offer of a programme of genetic testing and preventive treatment?

**Stage 2**. (the focus of this protocol)

Do the following factors relate to acceptability of a programme of genetic testing and preventive treatment in relatives of people with Paget's disease:

• Illness and emotional representations of Paget's disease;

• Treatment representations;

• Presentation of the disease in affected relative(s) (age at onset, impairment, activity limitations, participation restrictions; family history as a function of the number of affected relatives and the relationship to the Respondent);

• Respondent characteristics (age, gender and health);

• Cognitions about having a test and taking preventive treatment;

• The nature of the treatment offered.

By answering these research questions, this study will cast light on the feasibility of developing a programme of genetic testing and preventive treatment for individuals who are at high risk of developing Paget's disease because they carry *SQSTM1 *mutations. We do not yet know whether prophylactic bisphosphonate therapy would be effective in preventing Paget's disease, however, we envisage that the results of the present study would be used to inform the rationale of a randomised controlled trial to investigate the efficacy of prophylactic bisphosphonate therapy in people with *SQSTM1 *mutations. Such a study would form the basis of a future research project.

## Methods/Design

This research project has 2 stages.

### Stage 1

This part of the study is completed and addressed the first research question [39]. It aimed to identify factors that would influence involvement of relatives not known to have Paget's disease in a programme of genetic screening and preventive treatment. Stage 1 employed semi-structured interviews developed to elicit CS-SRM constructs and to form a basis for developing an Illness Perception Questionnaire [32] for individuals suffering from Paget's disease. It also employed Theory of Planned Behaviour (TPB)-based interviews [40], with their relatives who had not been diagnosed as suffering from Paget's disease to elicit beliefs about the consequences of the behaviours of accepting testing and treatment, about the views of important others regarding these behaviours and about whether these behaviours were easy or difficult for the respondent. The results of Stage 1 have been used to inform the development of this protocol and the questionnaire for Stage 2.

### Stage 2

This part of the study is the focus of this protocol. The Stage 2 has been reviewed and approved by Lothian Research Ethics Committee 1 (ref: 07/S1101/13). It will comprise a postal questionnaire study of people without Paget's disease but who are relatives of people with Paget's disease. This part of the study will address the second research question.

Two sets of individuals will be identified:

(1) Relatives of Paget's patients *with *a family history of Paget's disease (i.e. known history of Paget's disease in more than one blood relative);

(2) Relatives of Paget's patients *without *a known family history of Paget's disease (i.e. known history of Paget's disease in only one blood relative).

This stage aims to quantitatively assess the effect of a range of factors on the acceptability of a programme of genetic testing and preventive treatment in families with and without a family history of Paget's disease. The questionnaire is likely to consist of several measures: illness representations (as measured by the Brief Illness Perception Questionnaire [33]); treatment representations (as measured by a TPB-based questionnaire informed by Stage 1 [39]); a series of dependent variables exploring the acceptability of testing and treatment and the feasibility of a clinical trial of preventive treatment; questions collecting descriptive and demographic details; and questions exploring family environment and beliefs of other family members.

Data will also be collected from probands to describe the disease presentation and its distribution within a family.

The data from questionnaires completed by relatives will be analysed along with available data on members of the family with diagnosed Paget's disease (probands and other affected family members).

### Participants and recruitment

Individuals with Paget's disease will be identified, in the first instance, from two established cohorts: The PRISM trial and The Paget's Disease Family Register (PDFR). Probands and relatives from the PDFR cohort, and probands from the PRISM cohort, have previously given their consent to future contact regarding Paget's disease research (see Table 1 for additional details of how these individuals were approached).

**Table 1:** Approach to potential participants to invite them to participate in the GaP study

	**PRISM**	**PDFR**	**Edinburgh**	**NARPD**
**Probands**	Approach directly (after consultation with caring physician)	Approach directly (after consultation with caring physician)	Approach directly (after consultation with caring physician)	Approach directly
**Relatives**	Approach via proband	Approach directly (after consultation with caring physician)	Approach via proband	Approach via proband

We can identify in total, 254 probands with a family history of Paget's disease, and 1020 individuals without a family history of Paget's disease from these cohorts.

#### The PRISM Cohort

The PRISM cohort involves 1331 patients with Paget's disease from 39 collaborating centres ranging in size from 3 trial participants to approx 250 trial participants. Twenty of these centres have agreed to allow us to approach their patients for this study. We already know from the baseline evaluation that approximately 172 individuals in PRISM have a family history of Paget's disease, whereas 1020 individuals do not.

#### The Paget's Disease Family Register (PDFR)

We will also enrol participants with a family history of Paget's disease from the PDFR. This register currently comprises 49 probands and their affected and unaffected relatives from around the world. Families for this study have been enrolled from the UK [16]. These participants have already taken part in genetic mapping studies that resulted in the identification of *SQSTM1 *mutations as a cause of Paget's disease [3,8]. New families are being included on an ongoing basis and these figures are subject to change.

### Identifying and Contacting Participants

In the first instance, probands will be identified from both the PRISM and PDFR cohorts. For the PRISM cohort, all participants who have died, become unwell or who have withdrawn from the PRISM study are recorded on the PRISM database, and these participants will not be contacted for this study. For the PDFR cohort, every attempt will be made to avoid making inappropriate contact using a variety of methods. For example, where possible the status of probands will be checked with their caring physician/consultant.

If there is a high non-response from the PDFR and PRISM cohorts, we will identify probands from additional cohorts.

#### Contacting PRISM probands

Probands identified from the PRISM cohorts will be contacted by post. A letter will be sent to all probands together with a study information leaflet, inviting them to complete a questionnaire. Probands will also be asked to provide contact details of relatives over the age of 18 who may also like to participate. PRISM probands have previously been asked if they are happy to be contacted about new research projects, and only those agreeing to this will be contacted.

#### Contacting relatives of PRISM probands

A letter will be sent to relatives identified by probands together with a study information leaflet, inviting them to complete a questionnaire.

#### Contacting probands and relatives from the PDFR cohort

A letter will be sent to probands and relatives identified from the PDFR together with a study information leaflet, inviting them to complete a questionnaire.

### Questionnaire reminders

Non-response will be interpreted as a desire not to take part in the study. If no response has been received by the study office (Aberdeen) or Chief Investigator's office (Edinburgh) to the initial mailing, participants will not receive any subsequent questionnaire reminders or further contact from the study office.

### Sample Size

Given that most of these individuals have already engaged in research, we anticipate a high participation rate and estimate that 70% of probands will have living blood relatives and will be happy to contact their relatives. Based upon the experience of a recent study of familial hypercholestorolaemia [41] it is anticipated that an average of 2 relatives per participating proband will participate. The recommended minimum sample is calculated as 50 + 8 m, where m is the number of predictor variables [42]. This study has a total number of 26 predictor variables and the minimum sample size required is 258 relatives.

We anticipate that there will be a clustering effect of responses within families. The size of clustering effect is determined by the intra cluster correlation, and the sample size needs to be inflated to reflect this correlation [43]. We have no prior evidence of the possible size of this clustering effect, so assuming that the study has an average cluster size of 2 relatives per proband and that the correlation was 1 (the worst case scenario), the inflation factor would be 2. We therefore intend to recruit a minimum of 516 relatives to account for this effect. To achieve this it will be necessary to approach a minimum of 369 probands (accounting for 70% participation rate). We intend to approach a random sample of 200 probands with a known family history of Paget's disease, and 200 without.

### Exclusion criteria

Exclusion criteria include:

• Unable to read or write English; or

• Aged less than 18 years; or

• Known* limited life expectancy (under one year)

* The caveat of 'known' is used, as it may not be possible to determine limited life expectancy of relatives.

We have not made special arrangements for non-English speaking participants for two reasons:

• Paget's disease is extremely rare in non-Caucasians and therefore we anticipate that English will be the first language of virtually all, eligible individuals within the UK.

• The instruments used in this study (in particular the BIPQ and TPB) are sophisticated and sensitive tools for measuring an individual's perceptions of treatment and illness. Translating these measures into alternative languages necessitates re-validation of these instruments, which would require considerable investment of time and financial resources that are not available for this study.

Individuals with limited life expectancy are excluded from Stage 2 of the study as the process of completing a questionnaire may be cognitively and emotionally taxing for them.

A lower age limit has been imposed (minimum age of 18 years). Clinical studies suggest that people with *SQSTM1 *mutations develop Paget's disease from approximately 45 years of age onwards [7]. Clinical intuition would suggest that starting a programme of preventive treatment at age 40 years would therefore be appropriate. The clinical evidence to support this, however, is still lacking. In addition, it is likely that a psychologically 'acceptable' age for beginning preventive treatment will differ from that indicated by existing evidence. As such, we propose to include all adult relatives of probands to determine the effect of age of the acceptability of genetic testing, and preventive treatment.

### Consent Process

Although implied consent will be assumed if a subject completes and returns a questionnaire, we have additionally incorporated a brief consent form into the questionnaire to ensure that the following points are clearly understood:

• The general purpose of the study and questionnaire are understood;

• That the subject can withdraw from the study at any time and if they specifically request it, their data will be destroyed;

• The data is collected and securely stored by the Despatch Office in Edinburgh;

• We are not able to offer a genetic test or preventive treatment for Paget's disease as part of this study;

• That a summary of the results can be sent to them if they indicate agreement.

It is explicitly stated in the consent forms and questionnaires that participants can withdraw from the study at any time by contacting the study office and, if requested, their data will be destroyed.

The consent form incorporated into the questionnaires will also contain a tick box for participants to indicate whether they wish contact about future research projects concerning Paget's disease.

### Measures

A questionnaire will be sent to probands. This questionnaire will include:

• Details of disease presentation in the proband;

• An overview of the family structure;

• Details of relatives to contact.

A questionnaire will be sent to first and second-degree relatives of probands. This questionnaire will include specific measures of:

• All variables hypothesised to affect the likelihood of uptake of a programme of genetic testing and preventive treatment (potential explanatory variables; see section 6.1); and

• Dependent variables measuring the acceptability or intention to take up an offer of a programme of genetic testing and preventive treatment.

In addition, each section of the questionnaire will have a section of open questions.

### Potential explanatory variables

#### Illness and Emotional Representations

Illness and Emotional Representation will be assessed using the *Brief Illness Perception Questionnaire (BIPQ) *[33]) adapted for Paget's disease of bone. This is a very widely used predictive measure. Adaptations for specific conditions are part of the standard use of the questionnaire and Professor Weinman will provide critical feedback relating to the adapted measure before use. Appropriateness of items will also be tested for Discriminant Content Validity [44].

### Representations of Testing and Treatment Behaviours

Based on Stage 1 interviews, a questionnaire will be designed to assess beliefs about the behaviours involved in accepting a programme of genetic testing and preventive treatment, using TPB constructs and methods. The constructs are:

• *attitudes *to accepting a programme of genetic testing and preventive treatment;

• *subjective norm *refers to beliefs that important others would wish one to accept or not;

• *perceived behavioural control *refers to beliefs that a programme of genetic testing and treatment will be offered which they are able to accept; and

These constructs will be assessed using standard TPB methods [40] and based on beliefs (behavioural, normative and control) elicited in Stage 1. All measures will contain at least 3 items so that reliability can be assessed and will be subjected to psychometric analyses.

### Respondent characteristics

Age, gender, marital and employment status; relationship with affected person(s), number of affected relatives; self-assessed health as measured by SF-12 and self-assessed health on standard 4-point rating scale (excellent, good, fair, poor).

### Presentation of the disease in affected relative(s)

The following data will be collected from the proband: age at onset, previous fracture, previous bone surgery and the number of bones affected by Paget's disease. In addition, the SF-36 will give standardised measures of impairment, activity limitations, and participation restriction, based on previous work on the relationship between this measure and the ICF health components [45].

### Dependent variable: Acceptability of a programme of genetic testing and preventive treatment

Acceptability will be assessed through: intention to accept a test if offered and intention to accept preventive treatment if recommended by doctors. In addition, relatives' perceptions of the feasibility of taking preventive treatment will be measured through perceived behavioural control of particular forms of treatment. Intention and perceived behavioural control will be assessed using standard TPB methods [40].

Measures will be developed to assess *probability *of acceptance, using both numerical and verbal-rating scales and guidance from work on presenting probabilistic risk data. Using the TPB it is possible to measure intention and to assess the probability of engaging in the described behaviour. Information from Stage 1 interviews will be used for content, phrasing and context, and piloted in interviews until satisfactory. Participants will be specifically asked whether they would take up a programme of genetic testing and preventive treatment if it were to become available. In addition, we would specifically investigate the acceptability of the following options: preventive treatment options (tablet or infusion); and location of clinical intervention for preventive treatment (primary or secondary care; regional specialist centres or local.

### Data collection

Potential participants will be assigned a unique study number. This information will be held on a password-protected database/spreadsheet at the University of Edinburgh.

Questionnaires will be pre-labelled with these study numbers and posted to the corresponding potential participants. Questionnaires will be identifiable by the unique study number only.

Probands and relatives will be asked to complete and return the questionnaire in a reply-paid envelope. Help in completing the questionnaire will be available if required. If the patient is known to have difficulty writing (for example due to severe arthritis), is deaf or blind, special arrangements will be made for completion of the questionnaires.

Anne Langston, the Chief Investigator, and a data manager based at the Queen's Medical Research Institute, Edinburgh, will coordinate the administration of questionnaires and data management. Stage 2 will therefore be administrated from this site, and will be Sponsored by the University of Edinburgh.

Data will be returned by participants to the Edinburgh Office. There it will be entered onto an electronic database (password protected and stored on secure University server). An experienced data clerk will enter the data from questionnaires into this database. Anonymous data from this database will be transferred to the GaP study office at the University of Aberdeen for statistical analysis.

### Quality Assurance

Full verification checks will be undertaken at the time of data capture. A random 10% sample of data will be double entered to check accuracy. Extensive range and consistency checks will further enhance the quality of the data.

### Analysis

Stage 2 data analyses will involve psychometric assessment of developed and adapted measures. The main analyses will be carried out by a statistician in collaboration with Jill Francis, an expert in the psychometrics and analysis of theoretically based measures and analyses. and will involve multiple and logistic regression; this will be approached systematically, starting with analyses for each theoretical approach and then for each group of variables to identify significant predictors which will then be brought into an overall predictive equation.

Answers to open ended questions will be analysed to clarify content. The analysis of open questions will not involve a full qualitative analysis of all responses, but instead will be analysed as required to aid interpretation of quantitative results. Members of the research team will carry out this analysis. Detailed qualitative analysis may be carried out in the future and will be subject to separate funding and ethical approval.

### End of Study procedures

During the consent process for the study we will ascertain whether participants would like to be sent a report of the study results.

For those participants indicating that they would like study results, a short report will be prepared and circulated at the end of the study.

Questionnaires will be stored in locked filing cabinets until the study is complete, and will then be anonymised and archived for 20 years in accordance with Medical Research Council guidelines. The Chief Investigator, Anne Langston, will act as the Custodian of the data.

### Confidentiality

The Chief Investigator Office, based in the Queen's Medical Research Institute, Edinburgh, is responsible for the confidentiality of all study records. Potential participants will be assigned a unique study number. This information will be held on a password-protected database/spreadsheet at Edinburgh University. Questionnaires will be pre-labelled with these study numbers and posted to the corresponding potential participants. Questionnaires will be identifiable by the unique study number only. In accordance with Good Clinical Practice, and Institution codes of conduct, all data will be password protected against unauthorised access and stored in accordance with the Data Protection Act 1998. All stored data will be anonymised.

### Finance and Indemnity

The study is supported by a grant from the Medical Research Council (MRC). The University of Aberdeen holds the grant. Sponsorship responsibilities will be undertaken by the University of Edinburgh and NHS Lothian.

### Reporting and Dissemination

A summary of the results of the study will be prepared and distributed, not only to the appropriate funding body, but also to all participants (if they so wish). Results will be published in peer-reviewed journals to make the results available to both researchers on bone disease and researchers on health-related behaviours.

## Abbreviations

**CSSRM: **The Common Sense Self Regulation Model [29]. A psychological theory that aims to identify how people perceive their illness. **DNA: **Deoxyribonucleic acid. The substance that makes up genes. **Familial Paget's disease: **Paget's disease that affects more than one member of a family. **ICF: **International Classification of Function [34] identifies three health components: impairment of body structure and function; activity limitations; and participation restrictions. **IPQ: **The Illness Perceptions Questionnaire. This measures what people think about an illness in themselves or others. **IPQ-R: **The Illness Perceptions Questionnaire Revised [32]. This is a revised version of the Illness Perceptions Questionnaire that includes a measure of how understandable or confusing an illness is to people. **BIPQ: **The Brief Illness Perceptions Questionnaire [33]. This is a shortened version of the Illness Perceptions Questionnaire Revised. **MRC: **Medical Research Council. A UK government funded organisation that supports medical research [46]. **NARPD: **National Association for the Relief of Paget's Disease. A support group for people with Paget's disease, their family and carers [47]. **Non-familial Paget's disease: **Paget's disease that only affects one person in a family. **PDFR: **Paget's Disease Family Register. A study led by Prof Ralston, investigating the genetics of Paget's disease. **PRISM: **A trial led by Prof Ralston and co-ordinated by Dr Langston, investigating the treatment of Paget's disease. **Proband: **Initial person of contact within a family who has Paget's disease. **Relative: **A blood relative of a proband (i.e. brother, sister, mother, father, grandmother, grandfather, son, daughter, grandson, grand daughter, nephew, niece). **SF-36: **A questionnaire that measures general health, and quality of life. **SF-12: **A shortened version of the SF-36 questionnaire. **SQSTM1: **Sequestosome 1 [RNA NM_003900]. One of the genes identified that, when 'faulty', causes Paget's disease. **TPB: **The Theory of Planned Behaviour [40]. A psychological theory that aims to identify predictors of behaviour using a defined framework.

## Competing interests

Anne Langston received a small travel bursary in 2003 from The Alliance for better Bone Health, an alliance between Proctor & Gamble, and Sanofi Aventis, which are pharmaceutical companies who manufacture drugs used in the treatment of Paget's disease. She is also a Board Member and Trustee for the National Association for the Relief of Paget's Disease. Stuart Ralston acts as a consultant for Proctor & Gamble, Sanofi Aventis and Novartis, which are pharmaceutical companies who manufacture drugs used in the treatment of Paget's disease. Prof Ralston is also a Board Member and Trustee for the National Association for the Relief of Paget's Disease. Marilyn McCallum is the Chief Executive for the National Association for the Relief of Paget's Disease. There are no competing interests for the other authors.

## Authors' contributions

AL prepared and wrote the first draft of this protocol. CR contributed to the amendments and re-drafting of the protocol to achieve ethical approval. AL, MJ, MC, VE, JF, TM, GM, MMcC (consumer representative), ZM, KC (the lay advisor to the project) and SR designed the study and are grant holders. All authors have critically reviewed and approved the final manuscript.

## Pre-publication history

The pre-publication history for this paper can be accessed here:


